# The Role of Near-Infrared Spectroscopy (NIRS) in Neurological and Neurodegenerative Diseases as Support to Clinical Practice: An Overview of the Literature

**DOI:** 10.3390/diagnostics15070869

**Published:** 2025-03-28

**Authors:** Elvira Gjonaj, Caterina Formica, Emanuele Cartella, Nunzio Muscarà, Silvia Marino, Angelo Quartarone, Simona De Salvo

**Affiliations:** 1 IRCCS Centro Neurolesi Bonino Pulejo, 98123 Messina, Italy; elvira.gjonaj@irccsme.it (E.G.); nunzio.muscara@irccsme.it (N.M.); silvia.marino@irccsme.it (S.M.); angelo.quartarone@irccsme.it (A.Q.); simona.desalvo@irccsme.it (S.D.S.); 2 ASL Cagliari Ospedale Santissima Trinità, 09121 Cagliari, Italy; emanuele.cartella@libero.it

**Keywords:** near-infrared spectroscopy, neurological disorder, neurodegenerative disease, oxygenated hemoglobin, deoxygenated hemoglobin

## Abstract

Near-Infrared Spectroscopy (NIRS) is a non-invasive technique that measures the oxygenation variations of brain tissue in response to different stimuli. It has many advantages such as being easy to use, portable, and non-invasive. Several studies over the years have demonstrated the usefulness of NIRS in neurological and neurodegenerative diseases. NIRS remains relatively underutilized in clinical practice. The aim of this brief review was to describe the use of NIRS in neurological and neurodegenerative diseases and how its use can modify clinical, therapeutic, and rehabilitative approaches. A total of 54 relevant articles were selected from the PUBMED research database related to the diagnostic and prognostic role of fNIRS in the main neurological and neurodegenerative diseases; significant outcomes have been reported in a descriptive form with careful considerations. In addition, we excluded studies using fNIRS in co-registration with other neurophysiological techniques. The use of NIRS should be applied even in the field of neurological and neurodegenerative diseases; in dementia, NIRS can aid in differential diagnosis and predict possible evolutions from Mild Cognitive Impairment (MCI) to Alzheimer’s Disease (AD) stage; in stroke, it plays an important role especially in the post-acute phase, giving information about the patient’s chances of recovery; in Parkinson’s Disease (PD), the results showed the important role of cognitive aspects; in epilepsy, NIRS can localize the epileptic focus or potentially predict seizure onset.

## 1. Introduction

Near-Infrared Spectroscopy (NIRS) is a non-invasive technique that measures variations in the oxygenation of brain tissue, specifically the transition between oxygenated hemoglobin [oxy-Hb] and deoxygenated hemoglobin [deoxy-Hb], which results from neuronal activity (Functional NIRS [fNIRS]).

Near-infrared light (NIR) can penetrate biological tissue within an “optical window” due to the relative transparency of skin and bone. Although human tissue contains several chromophores that absorb NIR light, the absorption coefficient for oxy-Hb and deoxy-Hb is most effectively measured using a spectrum window from 700 to 2500 nm [1].

The absorption spectrum of hemoglobin changes depending on its oxygenation state, and this variation is crucial for understanding tissue hemodynamics. NIR light is emitted through an array of optical fibers (optodes), with the reflected signal collected by detectors placed on the scalp. These devices are located in different regions of the head because the hemodynamic response (vasodilatation and increase in blood volume) occurs in different spots of gray matter (cortical surface). Therefore, the back-scattered light, after absorption, provides information about chromophore concentrations in the tissue, which can be evaluated using the modified Beer–Lambert law (mBLL), a linear relationship between the absorbance and the concentration, molar absorption coefficient, and optical path length of a solution. In fact, it defines that the light attenuation through a medium (*A_λ_*) is proportional to the concentration of the light absorbers present in the substance (C_K_), the optical properties of the light absorber (*ε_K_*_(*λ*)_), and the optical path length traveled by the light beam (*L*). From this, the concentrations of the light absorbers in tissue are determined by processing the light attenuation. Among neuroimaging techniques, NIRS offers key advantages, such as ease of application, portability, low cost, safety, comfort, and good temporal resolution, although its lower spatial resolution and limited penetration depth are significant limitations. Despite its lower spatial resolution, shallow penetration depth (1.5–2 cm), and lack of data analysis standardization, NIRS is relatively resistant to motion artifacts compared to other neuroimaging techniques, resistant to interference, and compatible with electromagnetic fields [1].

Moreover, the optical nature of fNIRS enables it to be combined with other multimodal imaging techniques, such as High-Density Electroencephalogram (HD-EEG, 256 ch), Magnetic Resonance Imaging (MRI), and Transcranial Magnetic Stimulation (TMS). This multimodal approach allows for extensive data collection on neurovascular coupling, which is the relationship between cerebral blood flow and neural activity. In the last decade, the strengths of NIRS in the field of neuroscience have enhanced our knowledge about brain tissue variations in neurological disease. Recent advances have highlighted the functional synergy between NIRS and neuromodulation techniques, which can induce neuroplastic changes in the neural networks.

This overview of the literature explores the role and clinical application of fNIRS in common neurological and neurodegenerative disorders, its utility in consciousness disorders, and its evolving application in non-invasive neuromodulation [2].

This review aims to evaluate the clinical applications of fNIRS in neurological and neurodegenerative disorders, with a focus on its diagnostic, prognostic, and therapeutic potential.

## 2. Methods

A total of 54 papers were examined, focusing on the diagnostic and prognostic value of fNIRS in the main neurological and neurodegenerative diseases. In this literature overview, we conducted an analysis of NIRS applications to neurological diseases by focusing our attention on studies without co-registration. We selected studies from the 2000s onwards in the PUBMED database, originally using the following terms: “Near Infrared Spectroscopy”; “neurological disorder”; “neurodegenerative disease”; “oxygenated haemoglobin”; “deoxygenated haemoglobin”. Only articles that were tagged with these terms were included. We included articles written in English, and empirical research to contextualize our findings. We excluded those with co-registration in order to ensure the capability of the method itself, due to the lack of literature studies about co-registration approaches. Review articles, articles with no original data and no independent documents, and animal studies were excluded. Data fields included NIRS application, outcome measures, limitations, and conclusions. Papers were classified into four categories according to NIRS applications: diagnosis, prognosis, treatment, and correlation. This review is organized as follows: the fundamental principles of NIRS are outlined in the Introduction Section; the clinical applications of fNIRS for various neurological disorders—including dementia, stroke, epilepsy, Multiple Sclerosis, Parkinson’s Disease, Disorders of Consciousness, and sleep apnea—are presented in separate sections; and the significance of the outcomes, limitations, and future perspectives are discussed in the Discussion and Conclusion Sections.

## 3. Results

**Figure 1:**
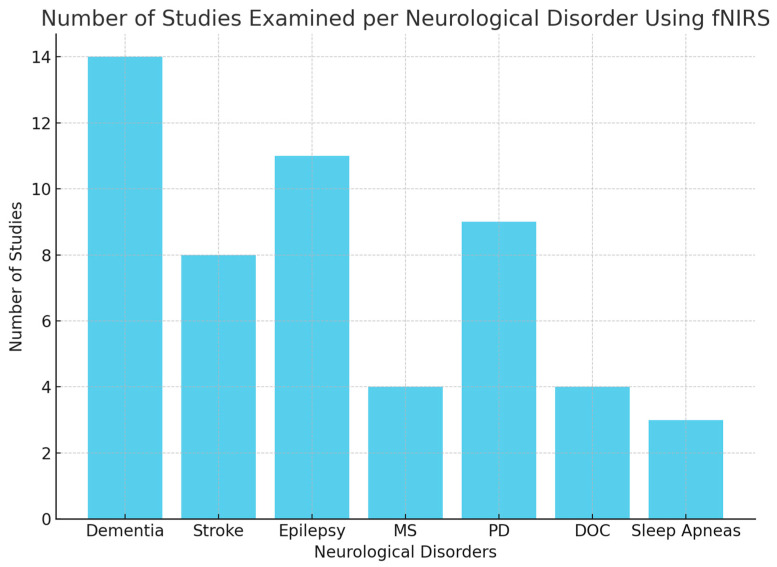
Distribution of studies across neurological disorders. Legend: MS = Multiple Sclerosis; PD = Parkinson’s Disease; DOCs = Disorders of Consciousness.

After article selection and screening, 54 studies were examined, without co-registration methods, divided for each neurological disorder that we discussed: dementia (14); stroke (8); epilepsy (11); MS (4); PD (9); DOCs (4); and sleep apneas (3) (Figure 1). The literature analysis yielded results which we have categorized into the following separate paragraphs based on the neurological diseases discussed.

### 3.1. Dementia

The prefrontal, frontal, temporal, and parietal areas were the most frequently studied and analyzed by fNIRS in dementia patients. Several studies employed different cognitive tasks during fNIRS acquisition to assess brain activity, including word retrieval, memory, motor control, verbal fluency, and visuospatial perception [3]. A recent review highlighted the usefulness of NIRS as an alternative method to analyze the different variations in oxygenation between patients with Alzheimer’s disease (AD) and patients with Mild Cognitive Impairment (MCI). This study showed a reduction in or interruption of cerebral perfusion in the frontal lobe during both resting and task execution states in AD and MCI patients compared to healthy controls [3]. The most commonly used cognitive task in dementia studies is the Verbal Fluency Task (VFT), which requires individuals to generate as many words as possible within a given time frame from a specific semantic category [4]. Fallgatter et al. [5] reported that AD patients showed less neural activation in the prefrontal cortex during the resting and task execution states compared to healthy subjects. Additionally, AD patients demonstrated greater activation in the right hemisphere, while healthy subjects only activated the left hemisphere. Arai et al. [6] described a decrease in the cortical activation of the frontal and parietal lobes in AD patients compared to healthy controls, while MCI patients showed a decrease in cortical activation only in the right parietal area. Hermann et al. [7] and Richter et al. [8] also found reduced oxy-Hb levels in the prefrontal cortex bilaterally in AD patients. Yeung et al. [9] published interesting findings that highlighted left frontal activation during tasks in healthy subjects, while MCI patients exhibited activation in both hemispheres, suggesting possible brain reorganization. Several studies have reported reduced activation in the frontal and temporal brain areas associated with a decrease in memory performance in MCI patients compared to healthy controls [10,11,12]. Doi et al. [13] focused on attention processes using a dual task approach, as well as a motor task (Dual-Task Walking, DTW), in MCI subjects. The results indicated an improvement in prefrontal cortex activity during DTW compared to single-task walking. In contrast, Zeller et al. [14] used visuospatial tasks (e.g., copying geometrical figures) to reveal parietal activations, showing decreased blood flow during task execution in AD patients. An interesting study by Kito et al. [15] compared depressed subjects, AD patients, and healthy controls, examining frontal and parietal cortex activations during VFT and visuospatial tasks. The study proposed that NIRS could be a valuable tool for misdiagnosis. In fact, the data revealed lower parietal cortex activation during the visuospatial task in depressed subjects, while no significant differences were found in verbal tasks. A recent study [16] highlighted NIRS as a promising method for distinguishing between subtypes of dementia: frontotemporal lobe dementia, Lewy body dementia, Parkinson’s Disease Dementia (PDD), and Alzheimer’s Disease (AD). Xi Mei and collaborators conducted an analysis on four patients during the VFT, working memory tasks, and resting states, comparing each patient’s responses during NIRS recording. The patient with frontotemporal dementia showed a reduced activation in the left frontotemporal and prefrontal lobes during VFT, while the Lewy body dementia patient exhibited asymmetry in the prefrontal lobes and low functional connectivity during the resting state. Moreover, the PDD patient showed lower excitability in the prefrontal cortex during VFT, but higher excitability during the working memory task. The AD patient exhibited reduced prefrontal and temporal activation during the working memory task. This study concluded that NIRS could aid in differential diagnosis by revealing different hemodynamic characteristics. A recent study [17] promoted an innovative approach combining NIRS with olfactory stimulation, applying a machine learning model. The results demonstrated notably high accuracy and sensitivity in identifying cognitive impairment in MCI and AD dementia. NIRS application could play an important role in order to support the differential diagnosis of dementia subtypes and predicting the conversion from MCI to AD. On the other hand, a distorted response of cerebrovascular reactivity seems to be correlated with compensation mechanisms in AD and MCI, which are conditions of protein A-beta accumulation that generate oxidative stress, resulting in reduced production of vasodilatory factors. Other studies, on the other hand, report a contradiction regarding compensatory responses already in the early stages of the disease in the reduction of hyperactivity or deactivation, resulting in subsequent protein aggregation and thus hypoactivation. Thus, indirectly, through the evaluation of parameters related to cerebral oxygenation, vasodilation, and constriction mechanisms, it is possible to measure or confirm that there is an underlying alteration due to the oxidative stress that characterizes these neurodegenerative pathologies [18].

### 3.2. Stroke

In recent years, several applications of NIRS have emerged, particularly in relation to stroke etiology and outcomes following an acute event. In the case of ischemic stroke, NIRS provides pivotal information on variations in cerebral blood flow and metabolism, which affect both the stroke-afflicted hemisphere and extend to the healthy contralateral side. By replacing the frontal montage of optical fibers with EEG electrode positions that include frontal, central, and parietal regions (according to the 10-10 international standard positioning system), temporal changes in the time domain are easier to detect. According to Giacalone et al. (2019) [19], patients with large vessel occlusion strokes had elevated concentrations of deoxygenated hemoglobin (Hb-DeoxyHb) and reduced oxygen saturation in both the ipsilateral and contralateral sides of the brain in comparison to controls. Interestingly, some areas of the stroke-affected hemisphere showed decreased oxygen saturation, while an ischemic recanalized area was also marked by increased Hb-DeoxyHb levels. This significant reduction in saturation, resulting from continuous oxygen consumption by surviving brain cells, provides strong evidence of brain metabolism in non-affected areas. Desaturation correlations observed by NIRS represent a novel parameter to better investigate acute stroke prognosis. While the use of NIRS in the hyperacute diagnostic stage may be limited—since it cannot reliably differentiate between ischemia and hemorrhage—it proves valuable in the subacute stage, after diagnosis and initial treatment. Continuous monitoring of oxygenation during this phase may provide early markers to identify clinical deterioration and prompt further clinical evaluation [20]. A recent review by Yang et al. [21] described the use of NIRS in stroke rehabilitation, in particular in assessing limb monitoring, motor learning, and recovery of the ipsilateral cortical function. Other studies have emphasized NIRS’ role in evaluating not only the stroke-affected areas but also their subsequent reorganization during rehabilitation [22]. Kato et al. [23] examined six patients with mild hemiparesis after middle cerebral artery infarction and found activation in both the contralateral cortex (as seen in healthy individuals) and the ipsilateral cortex during movement, suggesting compensatory changes in undamaged cortical tissue, including the unaffected hemisphere. In addition, NIRS can be a useful tool for assessing motor learning and neural mechanisms for gait and postural control [24]. Although few studies have specifically focused on stroke, these findings are promising and encourage further research, as NIRS could provide valuable insights into brain connectivity reorganization, contributing to rehabilitative recovery [25,26].

### 3.3. Epilepsy

Epilepsy is a neurological disorder characterized by significant hypersynchronization of neuronal populations, which spreads across brain regions. The epileptogenic zone is typically identified using electroencephalography (EEG), but more recently, NIRS has also been used to investigate frontotemporal epilepsy and explore network reorganization. Tung et al. (2021) [27] applied fNIRS to epilepsy patients during fluency tasks and analyzed brain connectivity. NIRS studies have focused on cortical oxygenation during seizures, localizing the epileptogenic area and distinguishing between different subtypes of epileptic fit, including focal and generalized seizures. Adelson et al. [28] monitored brain oxygenation changes before, during, and after epileptic attacks and found an increase in oxygenation several hours prior to the event. They also observed differences in oxygenation patterns between electroencephalographic crises (reduced oxy-Hb) and electro-clinical crises (increased oxy-Hb). Watenabe et al. [29] analyzed a group of drug-resistant epileptic patients and showed that, after inducing a seizure using bemegride injection, brain flow increased in the area of the epileptogenic focus. NIRS has proven useful in understanding the mechanism of epilepsy and possible misdiagnosis, by tracking oxygenation changes in both adults and children. Several studies have highlighted an increase in oxygenation only in the affected area in focal epilepsy, whereas the generalized form shows a decrease in oxygenation at the cortical level, demonstrating that blood supply occurs in all cortical areas with the presence of hypermetabolism [30,31,32,33,34]. Seyal et al. showed that NIRS could anticipate the onset of an epileptic seizure even a few minutes earlier (about 5 min) [35]. Over the years, NIRS has consolidated its role as an imaging tool for clinical application, particularly in preoperative functional assessment, such as evaluating language lateralization in drug-resistant epileptic patients [36]. It also provides specific information on language localization [37]. Importantly, unlike other methods, such as fMRI or MEG, NIRS has no contraindications and can be used with patients of any age [38].

### 3.4. Multiple Sclerosis

To date, the literature on the use of NIRS in MS is still limited, with only a few studies available. MS is known to be associated with a reduction in vascular hemoglobin levels, which can impact clinical disability. For instance, the study by Yang and Dunn found that NIRS recordings from the frontal cortex showed that 42% of tissue oxygen saturation (StO_2_) values in MS patients were significantly lower than those in the control group. This has led to the hypothesis that the presence of hypoxic areas in the brains of MS patients and alterations in microvascular circulation may contribute to neurological deterioration [39]. Due to the variability of MS disease stages, NIRS recordings could highlight controversial results about the alteration in the hemodynamic response variations. Contributing factors may include the challenges of integrating this technology into standardized clinical protocols and the need for further validation to determine the reliability and sensitivity of NIRS results across different stages of the disease. Furthermore, while NIRS has primarily been proposed as a tool to support clinical evaluation and monitor rehabilitative outcomes, available data remain fragmented. Studies such as those by Malagoni et al. [40] and Harp et al. [41], which examined changes in peripheral oxygenation in the gastrocnemius muscle at rest and during muscle activity, highlighted higher oxygen consumption during motor activity in MS patients, especially in those with reduced walking ability. The second study also noted a decrease in muscle metabolic rate and slower recovery after exertion. Another study by Reynolds et al. [42] investigated oxygenation changes in the right vastus lateralis muscle after 4 weeks of functional electrical stimulation (FES) cycling, showing improvements in oxygenation and muscle motor performance, particularly in patients with moderate to severe MS. While these findings suggest that NIRS could serve as a biomarker for peripheral adaptations related to mobility, disease progression, and motor therapy or rehabilitation programs, more comprehensive and longitudinal studies are needed to firmly establish its role in MS management.

### 3.5. Parkinson’s Disease

NIRS studies in Parkinson’s Disease (PD) evaluated the role of the prefrontal and frontal cortices during motor activities such as walking (normal walking or using electronic devices) and balance. However, there is currently a lack of standardization in participant recruitment and task methods [43]; in addition, the non-standardized data processing has limited the clinical relevance and interpretation of these studies. Mahoney et al. showed an improvement in oxygenation in the prefrontal cortex of advanced-stage PD patients during a motor and cognitive task that involved getting up and silently counting for 10 s [44]. The role of cognitive support in PD patients is well established, with several studies demonstrating its enhancement during the performance of motor tasks of different difficulty levels [43]. Maidan et al. [45] reported an increase in oxygenation in the frontal cortex during motor planning and information processing when simulating freezing episodes. Oxygenation levels increased when patients were warned about an impending freeze during the motor task, compared with neutral conditions. Additionally, increased oxygenation was found during normal walking, especially with obstacles, suggesting that the nature of the exercise influences cortical activity [46,47]. In this regard, differences in frontal cortex activation were also found between normal walking and treadmill walking with a decrease in oxy-Hb observed during mechanized gait [48,49]. Moreover, during deep brain stimulation of the globus pallidus internus (GPi) in PD patients, oxy-Hb levels increased in the frontal lobe through NIRS acquisition. Conversely, stimulation of the thalamic ventral intermedius nucleus (VIM) showed the presence of deoxy-Hb [50,51]. These results suggest a possible functional connection between the GPi and the frontal cortex, with the observed blood flow changes likely reflecting neuronal activations in these regions.

### 3.6. Disorders of Consciousness

Disorder of Consciousness (DOC) is a state of prolonged altered consciousness, which can be categorized into coma, vegetative state (VS), or minimally conscious state (MCS) based on neurobehavioral function. Patients awake but without external evidence of awareness are considered to be in a VS, while limited but clear evidence of interaction with the self and external environment, the presence of simple but reproducible behaviors are diagnosed as MCS [52]. Molteni and colleagues [53] studied motor and somatosensory cortices by NIRS in two MCS patients during sensory and motor stimulation. The data obtained showed the absence of activations during sensory stimulation, assuming a wrong protocol design or too low stimulation to activate the cortex (the patients did not communicate pain). However, motor stimulation (active and passive) led to an increase in oxy-Hb in the healthy side of the cortex, supporting MRI findings. Similar results were highlighted in a study by Kempny et al. [54], who evaluated brain function in DOC patients using a motor imagery task during the NIRS recording. They collected data from healthy controls performing both real and imagined motor movements. In control subjects, a greater oxy-Hb response was seen during actual movement compared to imagined movement. Comparing groups, they found a hemisphere effect, with greater depression of the oxy-Hb signal in the right hemisphere compared to the rest period for all three groups. MCS patients showed hemodynamic responses similar to the control group during the motor imaging task. In contrast, VS patients showed a different response: only one patient demonstrated similar feedback, while the rest of the group exhibited either reversed or absent reactions. Finally, Zhang et al. [55] assessed changes in cerebral blood volume in the prefrontal cortex in nine DOC patients with implanted spinal cord stimulation (SCS) devices, a promising neuromodulation treatment for DOCs. The results highlighted that intermittent SCS significantly improved blood volume fluctuations in the prefrontal cortex. Furthermore, patients with a favorable prognosis maintained higher blood flow levels in the prefrontal area after SCS and showed greater improvements in blood flow with stimulation. To date, NIRS studies in DOC patients remain limited, likely due to challenges such as the absence of patient communication during stimulations, the lack of standardized methodological criteria, and the severity of lesions in DOC patients. Nevertheless, NIRS represents a translatable imaging tool that could provide new insights into brain function in DOC patients, offering advantages such as its relatively low cost and patient tolerance in clinical settings.

### 3.7. Sleep Apneas

Obstructive sleep apnea syndrome (OSA) represents an important sleep disorder that impairs 9–38% of the population, from children to adults. Several studies were conducted to understand the hemodynamic consequences of this disorder. In particular, NIRS non-invasively evaluates changes in tissue oxygen saturation (SpO_2_) to detect cerebral circulation and changes in cerebral blood volume [56,57]. In 2003, Yamamoto and collaborators [57] applied NIRS to monitor hemoglobin concentrations continuously in the foreheads of 11 preterm infants. During 145 episodes of apneas, five groups were evaluated based on desaturation levels (ranging from <75% to ≥90%) to detect the SpO_2_ threshold at which cerebral circulation was impaired. They noticed that an SpO_2_ of <85% in preterm infants caused a reduction in blood pressure, decreased cerebral oxygenation, and significant changes in blood volume during apneic attacks. This threshold was correlated with circulation disturbances in low-birthweight infants, where autoregulation issues persist, differing from the autoregulation seen in adults [58].

## 4. Discussion

NIRS has been increasingly used in neuroimaging research due to its key advantages: (a) non-invasiveness, (b) minimal movement restrictions, (c) high temporal resolution, and (d) compatibility with other neuroimaging (e.g., MRI), neurophysiological (e.g., EEG), and neuromodulation (e.g., tDCS) techniques. These features make NIRS particularly valuable in clinical settings, especially for patients who are non-collaborative or difficult to assess with more traditional neuroimaging methods. Furthermore, NIRS is applicable to individuals of all ages and a broad range of pathologies, highlighting its versatility in clinical practice. Based on the results presented in this review, we believe that the use of NIRS will continue to play an expanding role in the management of neurological and neurodegenerative diseases. Specifically, in dementia, NIRS can aid in differential diagnosis and predict disease progression, such as the transition from MCI to AD. In stroke, it plays an important role, especially in the post-acute phase, by providing information on the patient’s potential for recovery. In PD, NIRS data indicate significant involvement of cognitive functions, pointing toward its utility in monitoring disease progression and cognitive decline. In epilepsy, NIRS can help locate the epileptic focus and even anticipate the onset of seizures, which could be a game-changer for early intervention. As we discussed previously, we did not find sufficient articles about the application of NIRS in MS and DOCs. The main limitations in the application of NIRS in MS and DOCs are likely technical challenges, patient variability, and a lack of comprehensive studies. In MS, reduced vascular hemoglobin and alterations in microvascular circulation make NIRS results difficult to interpret, and the integration of this technology into standardized clinical protocols is hindered by its limited sensitivity and reliability. In DOC patients, challenges arise from the difficulty in monitoring the brain of individuals who cannot communicate or show minimal responses, along with the lack of standardized methodological criteria. Additionally, in both conditions, the poorness of longitudinal studies and the heterogeneity of clinical presentations contribute to fragmented and inconclusive data. While NIRS is a powerful non-invasive tool for assessing brain function, it faces limitations that must be addressed for broader clinical adoption. One such limitation is the absence of standardized protocols (e.g., brain area, number of optodes, and task types). Moreover, the number of sources and detectors in the fNIRS setup determines the size of the brain area that can be measured, which is often limited to frontal/parietal regions. Similar restrictions on measurement also apply to brain depth. fNIRS is only able to detect NIR light that penetrates the first few centimeters of cortical tissue, making this one of fNIRS’ key limitations [59,60]. Additionally, motion artifacts during measurements can also lead to unreliable data, especially in clinical settings where patients may struggle to remain still for long periods. The presence of hair and variations in scalp thickness can interfere with sensor placement and signal quality, leading to inconsistent results between individuals. NIRS is also limited in its ability to capture hemodynamic changes accurately and does not directly measure neuronal activity, as the relationship between blood flow changes and neuronal firing is complex and varies between individuals. Calibration is required for each patient, and individual variability in tissue composition, skin tone, and skull thickness can affect signal interpretation. Additionally, while NIRS provides real-time data on oxygenation changes, its quantitative accuracy is lower than that of more established imaging techniques, limiting its precision for measuring subtle hemodynamic changes in clinical scenarios. This review presents a collection of studies on the application of NIRS in neurological and neurodegenerative diseases but makes no mention of co-registration with other neurophysiological methods. Only a recent study [17] promotes an innovative combination of NIRS with olfactory stimulation and machine learning models, showing high accuracy and sensitivity in identifying cognitive impairment in MCI or AD patients. A multimodal approach could be interesting to use in future prospective studies in neuroimaging research. It is also necessary to standardize the recording protocols for specific diseases and establish reference values (e.g., task duration and resting status) to ensure consistency and reliability across studies.

## 5. Conclusions

Based on current literature, fNIRS has proven to be a valuable non-invasive tool in clinical applications. This non-invasive technique offers the potential to analyze brain functions and detect abnormalities. It has already been used successfully to assess cerebral oxygenation and perfusion during cardiac and neurosurgery, as well as following head trauma. However, fNIRS remains complementary to imaging techniques like MRI, as its lower spatial resolution limits its standalone diagnostic capability. Looking forward, the constant evolution of fNIRS technology in spatial resolution may facilitate its integration into clinical practice, providing deeper insights into the mechanisms underlying brain pathology and physiology. This will enable the development of more effective strategies to improve patients’ outcomes. Standardization of protocols and integration with other neuroimaging modalities will be key in maximizing the clinical utility of fNIRS.

## Data Availability

The manuscript does not support data because it is an overview of global literature with careful considerations.

## Abbreviations

The following abbreviations are used in this manuscript:

NIRS	Near-Infrared Spectroscopy
fNIRS	Functional Near-Infrared Spectroscopy
Oxy-HB	Oxygenated hemoglobin
Deoxy-Hb	Deoxygenated hemoglobin
mBLL	Modified Beer–Lambert law
HD-EEG	High-Density Electroencephalogram
EEG	Electroencephalography
MRI	Magnetic Resonance Imaging
TMS	Transcranial Magnetic Stimulation
AD	Alzheimer’s Disease
MCI	Mild Cognitive Impairment
VFT	Verbal Fluency Task
DTW	Dual-Task Walking
PD	Parkinson’s Disease
PDD	Parkinson’s Disease Dementia
MS	Multiple Sclerosis
StO_2_	Tissue oxygen saturation
FES	Functional electrical stimulation
GPi	Globus pallidus internus
VIM	Ventralis Intermedium Nucleus
DOC	Disorder of Consciousness
VS	Vegetative state
MCS	Minimally conscious state
SCS	Spinal cord stimulation
OSA	Obstructive sleep apnea
SpO_2_	Saturation of peripheral oxygen
tDCS	Transcranial Direct Current Stimulation
